# Ecological properties of shoot- and single seeds in a hardwood, *Zelkova serrata*

**DOI:** 10.1016/j.dib.2018.04.101

**Published:** 2018-05-01

**Authors:** Hiroki Oyama, Osamu Fuse, Hiroshi Tomimatsu, Kenji Seiwa

**Affiliations:** a Tohoku University, Japan; b Miyagi prefectural Forestry Technology Institute, Japan; c Yamagata University, Japan

## Abstract

The data presented in this paper is supporting the research article　“Variable seed behavior increases recruitment success of a hardwood tree, *Zelkova serrata*, in spatially heterogeneous forest environments” (Oyama et al., 2018) [1]. We provided the data of several ecological properties of the two types of the seeds (i.e. shoot seeds vs. single seeds) with distinctly different dispersal behaviors. We provide data of terminal velocity, which was measured by releasing 50 replicates of each seed type from a height of 5.0 m in dead air space in a gymnasium. We also show the data of germination cue [i.e. red:far-red (R:FR) ratios], which was examined in plant growth chambers that received three distinct R:FR ratios (0.1, 0.4, and 1.0; 16 h photoperiod) or no illumination. Further, we show the data of the rates of multi-locus outcrossing rates and biparental inbreeding in each of single- and shoot seeds. The mating system parameters were estimated by assaying a total of 80 shoot seeds and 70 single seeds that were randomly collected from Parent 1 and Parent 2 for five microsatellite loci. Finally, we show the data of hemispherical canopy photographs, which were taken at different distance from the adults using a digital camera equipped with a fisheye lens.

**Specifications table**

**Table:** 

Subject area	*Biology*
More specific subject area	*Forest Ecology*
Type of data	*Figures and Tables*
How data was acquired	*Seed germination experiment in laboratory, seed dispersal experiment, Molecular analysis,hemispherical canopy photographs*
Data format	*Analyzed*
Experimental factors	*Brief description of any pretreatment of samples*
Experimental features	*Very brief experimental description*
Data source location	*Miyagi, Japan*
Data accessibility	*With this article*
Related research article	Oyama, H., Fuse, O., Tomimatsu, H. and Seiwa, K. Variable seed behavior increases recruitment success of a hardwood tree, *Zelkova serrata*, in spatially heterogeneous forest environments, For. Ecol. Manag. 415–416 (2018) 1–9.

**Value of the data**

• The terminal velocity (cm s^−1^) as a measure of wind dispersal, assumes that the slower the rate of fall, the greater the dispersal potential of individuals seeds.
• Differences in seed germination cue (i.e. red:far-red ratios) between the seed types show the potential ability in either gap detection or persistent seed bank under shaded forest understory.
• Outcrossing rate and biparental inbreeding show the genetic background of seed heteromorphism in the context of seed dispersal ability and consequent juvenile performance.
• Measurements of hemispherical canopy photographs at 5 m intervals (2.5, 7.5, 12.5, 17.5, 22.5, and 27.5 m from an adult) along the centerline of each circular sector for each adult provide the data of light conditions of the seedlings.

## 1 Data

**Fig. 1:**
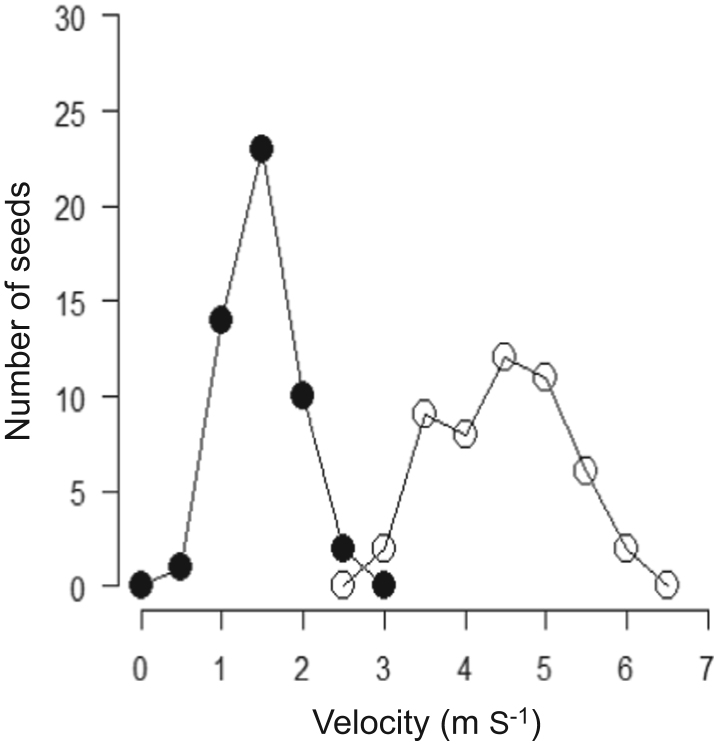
Terminal velocity (m s^−1^) of single-seeds (◯) and shoot-seeds (•).

**Fig. 2:**
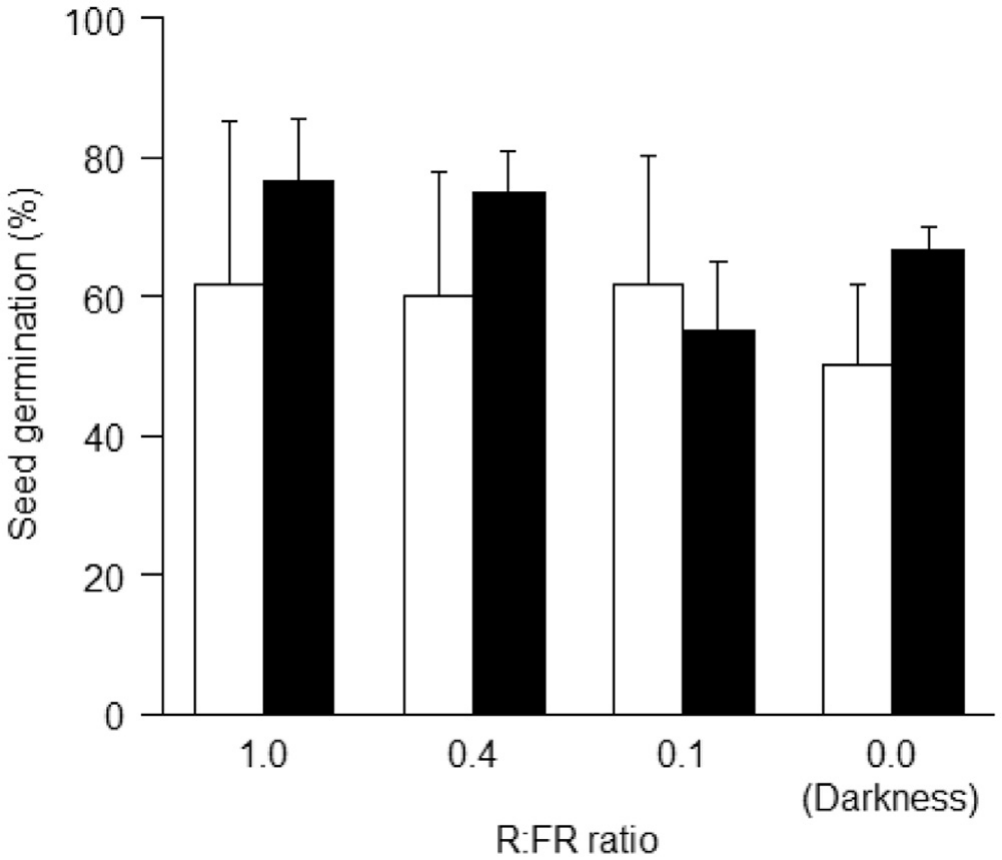
Percentage of seed germination under different red:far-red (R:FR) ratios in the laboratory in *Zelkova serrata.* White and black bars indicate single seeds and shoot seeds, respectively.

**Fig. 3:**
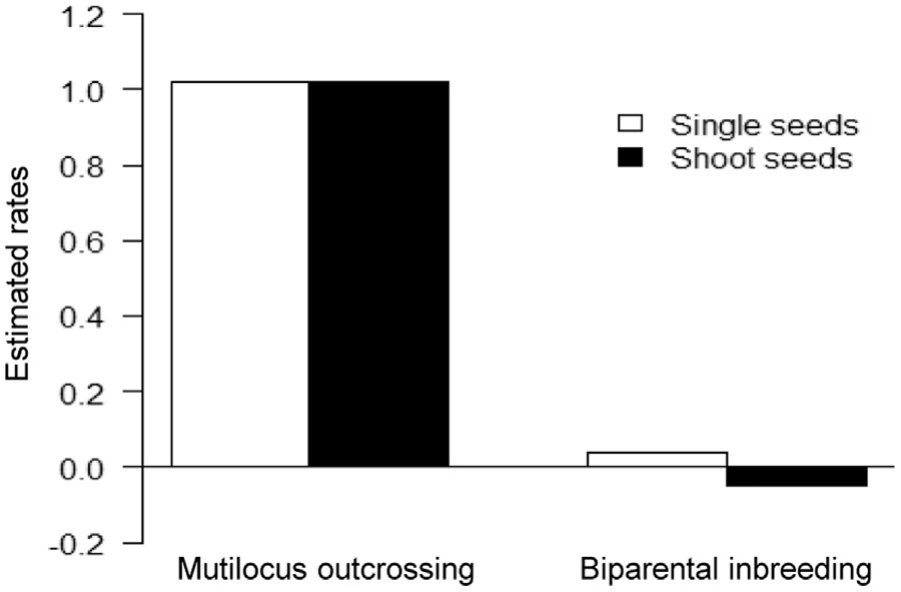
Rates of multi-locus outcrossing rates and biparental inbreeding for single- and shoot-seeds in *Zelkova serrata*.

**Fig. 4:**
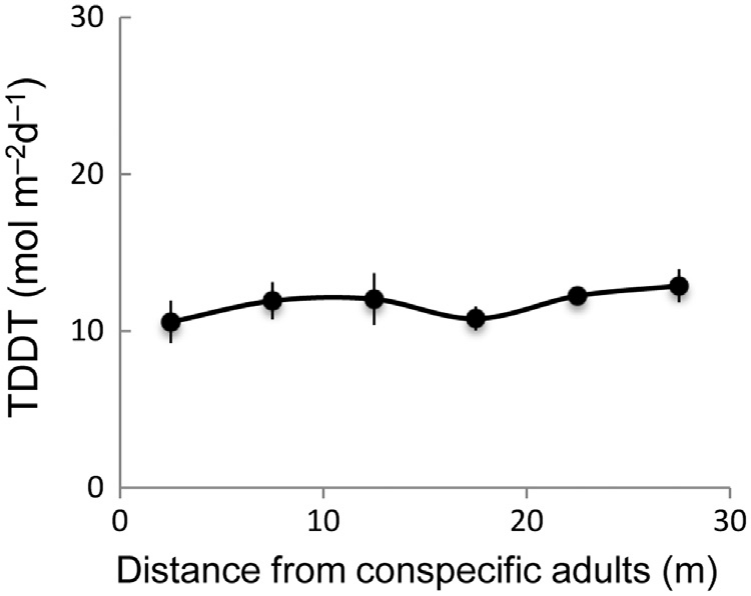
Total direct and diffuse transmittances (TDDT; mol m^−2^ d^−1^) at different distances from the adults of *Zelkova serrata*.

The data presented here was the basis for the research article by Oyama et al. [1]. We provided the data of terminal velocity (Fig. 1, Table 1), seed germination cues (Fig. 2, Table 2), rates of multi-locus outcrossing rates and biparental inbreeding (Fig. 3) of the two types of the seeds (i.e. shoot seeds vs. single seeds). Data of hemispherical canopy photographs (Fig. 4) was also provided.

### 1.1 Terminal velocity

See Fig. 1 and Table 1.

**Table 1:** Results of GLM analysis in the difference of terminal velocity between seed type (single- vs. shoot-seeds) in *Zelkova serrata*.

	Estimate	*T* value	SE
Seed type	3.024	24.08***	0.126

*** *P*<0.001

### 1.2 Seed germination cues

See Fig. 2 and Table 2.

**Table 2:** Results of GLMM analysis of the effects of seed type (single- vs. shoot-seeds), R: FR ratio (four different R:FR ratios) and their interaction (seed type x R:FR ratio) on percentage of seed germination in laboratory in *Zelkova serrata*.

	Estimate	*T* value	SE
Seed type	−0.274	−1.02	0.267
R: FR ratio	0.574	1.68	0.341
Seed type × R: FR ratio	0.124	0.26	0.474

### 1.3 Rates of multi-locus outcrossing rates and biparental inbreeding

See Fig. 3.

### 1.4 Hemispherical canopy photographs

See Fig. 4 and Table 3.

**Table 3:** Results of GLMM analysis of the effects of distance from adults on light conditions (TDDT) of plots observed the seedlings of *Zelkova serrata*.

		Estimate	*T*-value	SE
Light condition (TDDT, mol m^−2^ d^−1^)	Distance	0.021	1.248	0.017

## 2 Experimental design, materials, and methods

Data of terminal velocity was obtained by the following measurement. We released 50 replicates of each seed type from a height of 5.0 m in dead air space in a gymnasium. We assumed that fruits reached terminal velocity quickly [3] thus, the fall time was inversely related to terminal velocity. GLM analyses were conducted to test for the effects of seed type (single vs. shoot seeds) on terminal velocity using a normal distribution. The measurement of the terminal velocity (cm s^−1^) as a measure of wind dispersal, assuming that the slower the rate of fall, the greater the dispersal potential [2].

The data of germination cues was obtained as follows. The effects of red:far-red (R:FR) ratios on seed germination were examined in plant growth chambers (MTI-202B; Tokyo Rikakikai, Tokyo, Japan) that received three distinct R:FR ratios (0.1, 0.4, and 1.0; 16 h photoperiod) or no illumination. Light was provided by stick-type light-emitting diode (stick-LED) lamps (Tokyo Rikakikai) that emitted most of their light radially. We arranged 12 stick-R (wavelength: 660 nm) and 13 stick-FR (peak wavelength: 740 nm) LEDs either homogeneously or alternately on panels (width×depth×height: 314 mm×258 mm×60 mm) attached to the ceilings of the growth chambers. The R:FR ratio of the radiation emitted by the two types of stick LEDs was modified using a direct current power supply (PR18-5AYE type; TEXIO Technology, Tokyo, Japan). Light levels were measured using a spectroradiometer (MS-720; Eiko Instruments). Photosynthetic photon flux density (PPFD) levels were 2.5, 8.7, and 25.0 μmol m^−2^ s^−1^ at R:FR ratios of 0.1, 0.4, and 1.0, respectively. Four growth chambers were used, and each received one of the three R:FR ratios (0.1, 0.4, and 1.0) or no light (dark) at all. The seeds in each chamber experienced a constant temperature of 20 °C. Before the start of the germination experiments, seeds were disinfected in sodium hypochlorite (1%) for 30 min. Four Petri dishes (diameter: 70 mm×15 mm) were placed in each growth chamber, and two replicate dishes contained 20 seeds of either single or shoot seeds. All of the seeds were collected from 26 September to 16 November. Seeds were also placed on two sheets of filter paper in four Petri dishes in a dark room. The filter paper was moistened with distilled water. All of the dishes were wrapped in transparent plastic wrap to maintain optimal moisture content; the Petri dishes given the dark treatment were wrapped in silver-lined paper to prevent the penetration of light. Seed manipulations were performed in dim light (<0.01 μmol m^−2^ s^−1^) in a light-tight box. Germinated seeds were counted and removed, and distilled water was added at weekly intervals. These experiments continued until no germination was observed for 2 weeks in any of the treatments. To test for the effects of seed type and R:FR ratio on the percentage of seed germination, we used GLMM analyses, assuming a binomial distribution. Seed type and R:FR ratio were fixed effects, and container origin (Petri dishes) was set as a random effect.

The rates of multi-locus outcrossing and biparental inbreeding for single- and shoot-seeds in *Zelkova serrata* were estimated by the following methods. We estimated mating system parameters by assaying a total of 80 shoot seeds and 70 single seeds that were randomly collected from Parent 1 and Parent 2 for five microsatellite loci (*bczs143a*, *bczs144a*, *bczs157c*, *bczs184a*, and *bczs241a* [4]). Total DNA was extracted from seed embryos using SDS-proteinase K treatment [5], [6]. Each seed embryo was crushed in a 1.5 mL tube using a toothpick; subsequently, 10 μL reaction buffer (20 mM Tris–HCl buffer [pH 8.0], 100 mM KCl, 2 mM MgCl_2_, 0.01% proteinase K, and 0.1% SDS) was added to the tube, and the tube was incubated at 37 °C for 60 min and then heated at 95 °C for 10 min. The resulting extracts were directly used as DNA templates for subsequent polymerase chain reactions (PCRs). We also extracted DNA from the dried leaves of parent trees using a DNeasy Plant Mini Kit (Qiagen, Hilden, Germany). PCRs were performed separately for each locus in a total volume of 5 μL containing 0.5 μL template DNA, 1× Qiagen Fast Cycling PCR Master Mix (Qiagen), and 0.5 μM each primer. PCR amplifications were performed using an iCycler (Bio-Rad, Hercules, CA, USA) under the following conditions: initial activation at 95 °C for 5 min, followed by 35 cycles of denaturation at 96 °C for 5 s, annealing at 56 °C for 5 s, primer extension at 72 °C for 9 s, followed by a final extension step at 72 °C for 1 min. PCR products for all loci were mixed together with 10 μL Hi-Di formamide and 0.15 μL GeneScan 500 LIZ Size Standard and electrophoresed on an ABI PRISM 3130xl Genetic Analyzer (Applied Biosystems, Foster City, CA, USA). The resulting data were analyzed using GeneMapper 4.0 (Applied Biosystems).

We computed average multi-locus and single-locus outcrossing rates (*t*_*m*_ and *t*_*s*_, respectively) using MLTR, which estimates these mating system parameters for different groups of plants by assuming that pollen allele frequencies are homogenous among groups [7]. Newton–Raphson iteration was used to determine maximum likelihood estimates. Because single-locus estimates include mating between relatives, the difference between multi-locus and single-locus estimates can be used to infer the level of biparental inbreeding. Multi-locus outcrossing rates (*t*_*m*_) and the level of biparental inbreeding (*t*_*m*_−*t*_*s*_) were compared qualitatively between shoot and single seeds, as the small number of progeny arrays (i.e., two arrays) did not allow the bootstrapping necessary to obtain standard errors of the estimates.

Hemispherical canopy photographs were taken using a digital camera equipped with a fisheye lens (Nikon FC-E8 0.21×) on 5 September 2012 at a height of 0.8 m from the ground. The measurements were conducted at 5 m intervals (2.5, 7.5, 12.5, 17.5, 22.5, and 27.5 m from an adult) along the centerline of each circular sector for each adult. Total direct and diffuse transmittances (TDDT; mol m^−2^ d^−1^) were calculated using GLA for analyzing hemispherical images [8]. The effects of distance from adults on TDDT were analyzed using GLMM, assuming a normal distribution. Adult locality was set as a random effect.
